# Dynamic Equilibrium at the HCOOH-Saturated TiO_2_(110)–Water Interface

**DOI:** 10.1021/acs.jpclett.2c03788

**Published:** 2023-03-23

**Authors:** Fernanda Brandalise Nunes, Nicolò Comini, J. Trey Diulus, Thomas Huthwelker, Marcella Iannuzzi, Jürg Osterwalder, Zbynek Novotny

**Affiliations:** † Department of Chemistry, University of Zürich, CH-8057 Zürich, Switzerland; ‡ Department of Physics, University of Zürich, CH-8057 Zürich, Switzerland; § Swiss Light Source, Paul Scherrer Institut, CH-5232 Villigen PSI, Switzerland; ∥ Empa, Swiss Federal Laboratories for Materials Science and Technology, Laboratory for Joining Technologies and Corrosion, CH-8600 Dübendorf, Switzerland

## Abstract

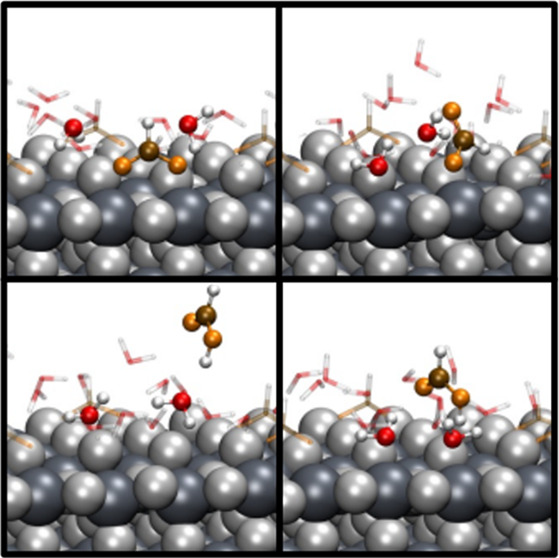

Carboxylic acids bind to titanium dioxide (TiO_2_) dissociatively, forming surface superstructures that give rise to a (2 × 1) pattern detected by low-energy electron diffraction. Exposing this system to water, however, leads to a loss of the highly ordered surface structure. The formate-covered surface was investigated by a combination of diffraction and spectroscopy techniques, together with static and dynamic ab initio simulations, with the conclusion that a dynamic equilibrium exists between adsorbed formic acid and water molecules. This equilibrium process is an important factor for obtaining a better understanding of controlling the self-cleaning properties of TiO_2_, because the formic acid monolayer is responsible for the amphiphilic character of the surface.

Titanium dioxide (TiO_2_) is well-known for its photocatalytic properties,^1^ although its large band gap requires irradiation in the ultraviolet range. An interesting example is its self-cleaning effect^2^ or the hydrophobic/hydrophilic switching of the rutile(110) surface.^3^ Ultraviolet (UV) irradiation in the presence of molecular oxygen and water leads to both high hydrophilicity and oleophilicity, as determined by contact angle measurement. Storage in the dark progressively leads to a hydrophobic behavior over a period of days.^3^ Further studies investigated how this change in wettability is related to the presence of contaminants.^4^ Upon exposure of a TiO_2_(110) single crystal to molecular oxygen with different partial pressures of hexane, it was possible to observe a sudden decrease in the contact angle of a pure water droplet after UV irradiation over a certain time span. Higher hexane concentrations correspond to longer incubation times, while in the absence of hexane, the contact angle decrease is immediate. On the contrary, in the absence of oxygen, this decrease does not occur. These observations reveal the role of organic contaminants and their photocatalyzed oxidation on the surface’s hydrophobicity.^4^

The specific interaction of water with the TiO_2_(110) surface has been studied extensively, distinguishing among conditions for molecular and dissociative adsorption.^5−7^ Water is experimentally found to dissociate at oxygen vacancies, forming a pair of bridging hydroxyls. On a defect-free TiO_2_(110) surface, a water molecule can dissociate forming a terminal and bridging hydroxyl pair, although molecularly bound water is slightly energetically preferred over the deprotonated configuration.^6^ Only recently have well-defined single crystals been exposed to liquid water, followed by characterization using a surface science approach. The presence of an ultrathin water layer can change the reaction energetics significantly, as was previously demonstrated for a spontaneous reaction of atmospheric CO_2_ with the nanoscale film of H_2_O adsorbed to the rutile(110) surface.^8^ Early studies on a clean TiO_2_(110) surface suggested that liquid water could form a (2 × 1) periodicity due to the ordered array of hydroxyl molecules with a liquid water in the second layer.^9^ Later scanning tunneling microscopy (STM) and X-ray photoelectron spectroscopy (XPS) experiments demonstrated that ultrapure, oxygen-free water itself is not responsible for the observed surface (2 × 1) periodicity.^10^ In fact, the presence of formic or acetic acid molecules, which are always present at low concentrations in air, dominates the process and is expected to affect the wetting properties of the surface. Carboxylic acids adsorb readily on the TiO_2_(110) surface, saturating the surface even upon interaction with liquid water. Still, a detailed knowledge of the interaction of water with a surface decorated with carboxylic acids, such as formic acid, is missing.

The dissociative adsorption of formic acid leads to a (2 × 1) reconstruction of the surface.^11^ Formate (HCOO^–^) binds through both of its oxygen atoms with two adjacent, 5-fold-coordinated titanium atoms. During this adsorption process, the dissociated proton attaches to a nearby bridging oxygen, forming a bridging hydroxyl. While different adsorption geometries are possible,^12^ this is the most favorable one,^13^ as was previously verified by X-ray photoelectron diffraction (XPD),^14^ STM,^15,16^ and infrared reflection–absorption spectroscopy.^12^

In the work presented here, we further investigate the co-adsorption of formate and water molecules at the TiO_2_(110) surface. Our results show that despite the higher stability of formate adsorption, the presence of a small amount of water at a finite temperature is sufficient to induce the rearrangement of the adsorbate superstructure and the dynamical exchange between water and HCOOH at exposed titanium sites. This finding is imperative for further understanding the surface structure stability in ambient environments, and also for controlling the self-cleaning properties of TiO_2_, because the high solubility of the surface formate in water could enable the water sheeting action under rinsing.

To better understand the co-adsorption of water and formate at a single-crystal TiO_2_(110) rutile surface, a combination of low-energy electron diffraction (LEED) and ambient-pressure XPS (APXPS) experiments, together with a series of static and dynamic density functional theory (DFT)-based simulations, were performed. TiO_2_(110) surfaces were saturated with formate using a 50 Langmuir (L) dose of HCOOH, as manifested by LEED patterns showing the typical (2 × 1) reconstruction. Figure 1 shows carbon 1s XPS spectra collected in high vacuum (HV, 10^–7^ mbar) prior to and after the sample was dipped into liquid water, colored black and red, respectively. At no time during the course of this experiment was the sample exposed to air, only to water vapor (25 mbar pressure) held in static equilibrium with liquid water during the transfer. The spectra are dominated by an “adventitious” carbon peak at 285.0 eV, related to C–C bonds, which is unavoidable in this pressure range. A detailed analysis of the C 1s region under similar experimental conditions can be found in ref (17). Before exposure of water, a distinctive peak is observed at 289.3 eV, commonly associated with surface formate.^10^ Calculating the formate coverage with a thin film model^18^ yields an approximate 0.5 ML coverage, consistent with a fully saturated TiO_2_(110) surface, while the peak area of the “adventitious” carbon peak is 6.1 ± 0.7 times higher than the formate peak. The saturation coverage of formate indicates that the “adventitious” carbon does not uniformly cover the surface but is rather present in the form of large clusters. Because most X-ray photons penetrate through these clusters due to the glancing incidence angle, they disproportionately contribute the C 1s signal located at 285 eV in Figure 1. After exposure to water, the 289.3 eV formate peak is replaced by a broader and weaker shoulder that is commonly present as the level of “adventitious” carbon contamination increases (having a 17 ± 2 times larger area compared to the initial formate peak).^17^ This suggests that adsorbed formate molecules are displaced upon exposure to water vapor. While the feasibility of rinsing the ordered monolayers due to their high solubility in water was reported previously,^10,19^ XPS also highlighted the use of a high-intensity synchrotron X-ray beam is inadequate. Much of the “adventitious” carbon buildup is a result of the exposure to the X-ray beam.^17^ In addition, the secondary electrons produced by the XPS technique are known to induce a rapid degradation of the ordered formate overlayer.^20^

**Figure 1:**
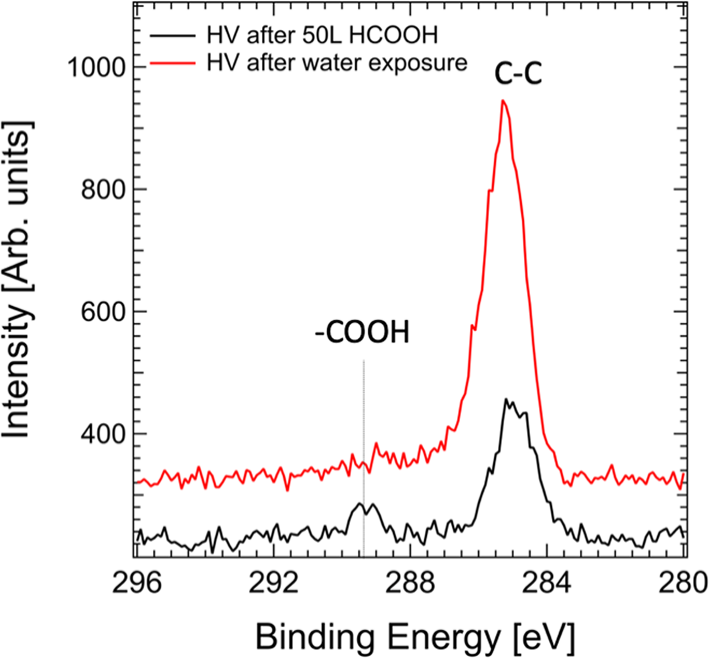
XPS spectra of a TiO_2_(110) surface in the C 1s region acquired at a pressure of 10^–7^ mbar (termed HV). The black spectrum was acquired immediately after the surface had been saturated with formic acid (50 L dose), and the red spectrum was acquired after the sample was dipped in liquid water, followed by transfer via water vapor (25 mbar) to HV without exposure to air. The dominant peak at 285.0 eV reflects the increase in the level of “adventitious” carbon contamination (C–C and C–H bonds), while the distinctive peak appearing at 289.3 eV after HCOOH dosing is much reduced after exposure to water. The former peak appears to be slightly shifted as the contamination does not represent a well-defined carbon species.^21^

To investigate this phenomenon more thoroughly, formic acid-saturated surfaces of TiO_2_(110) were exposed to increasing doses of water vapor at room temperature (297 ± 2 K). Samples were placed in the APXPS analysis chamber where pumping was interrupted by closing a gate valve as the chamber was backfilled with water vapor to a desired pressure. After a chosen time, the gate valve was reopened to quickly return to HV where LEED patterns were measured in the preparation chamber using commercial low-current LEED optics to avoid electron-induced degradation of the formate overlayer caused by the measurement.^20^ To reduce the effect of carbon contamination (as shown in Figure 1), the analysis chamber was exposed to repeated cycles of exposure to water (millibar range) followed by pumping to HV, which progressively reduced the amount of “adventitious” carbon.^17^ This allows the observation of how different doses of water interact with the ordered formate.

Figure 2 shows LEED patterns acquired in these experiments, from which one can observe that the (2 × 1) spots due to the ordered formate layer become progressively fainter as the total water dose increases. The intensity ratios of the (2 × 1) versus the (1 × 1) diffraction spots were quantified by evaluating the spot brightness. The results are plotted as a function of water vapor dose in Figure 3, showing roughly a linear-log behavior. The data display a decrease in LEED spot intensities corresponding to an ordered formate layer as the dose of gas phase water increases. During the interaction between the sample and water vapor, the order of the formate film appears to progressively weaken and then eventually vanish. This effect is not induced solely by an increase in the level of surface carbon contamination due to the increased pressure, as the (1 × 1) spots remain robust.

**Figure 2:**
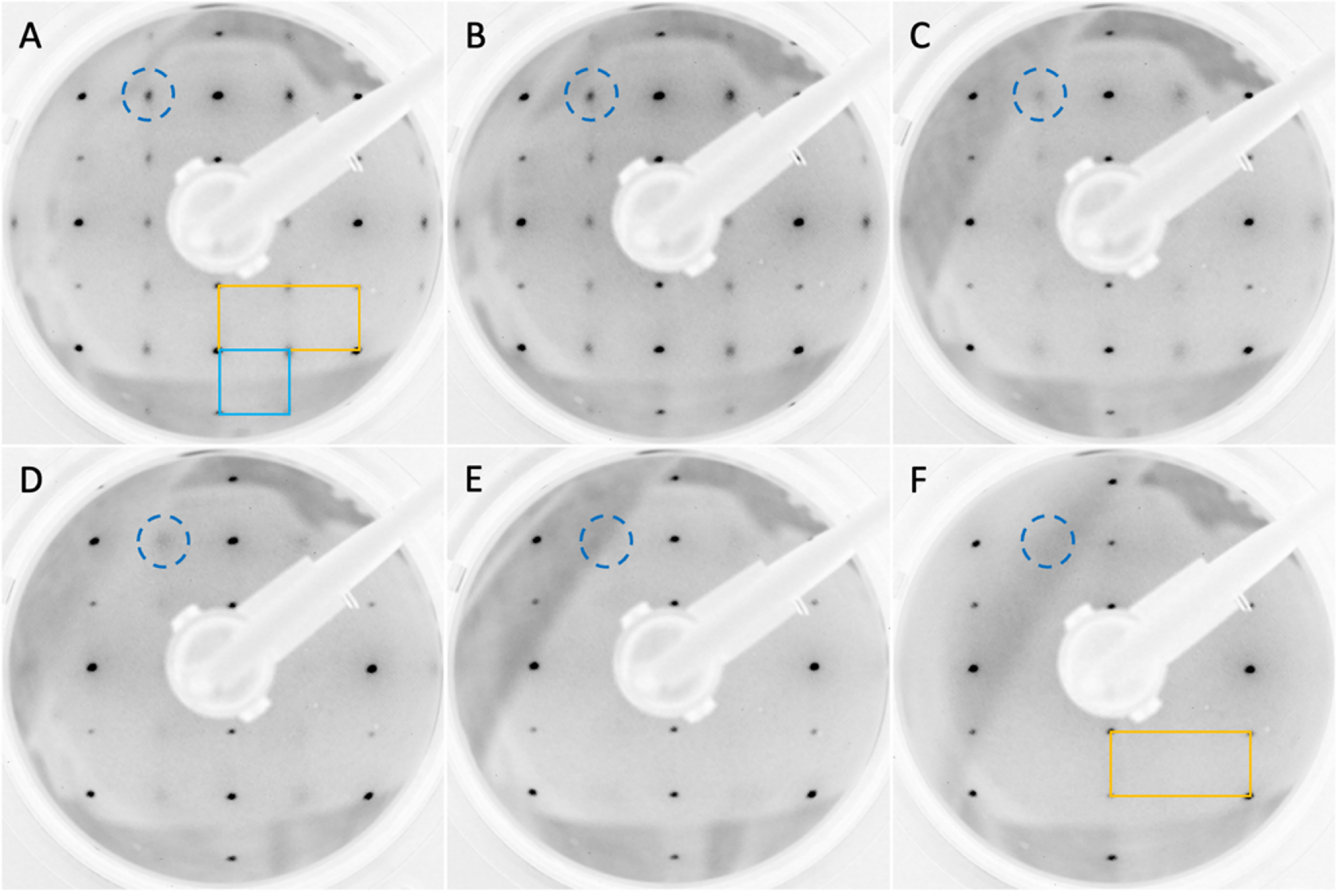
Processed LEED images of a TiO_2_(110) surface saturated with HCOO^–^, acquired after exposure to various doses of water vapor. (A) Sample as prepared, after dosing with 50 L of HCOOH. The yellow rectangle indicates the original (1 × 1) surface structure, while the cyan square represents the (2 × 1) reconstruction induced by the ordered layer of formate. (B) After 5 × 10^–6^ mbar water vapor exposure for 10 min (2.25 × 10^3^ L). (C) After 1 × 10^–3^ mbar for 10 min (4.5 × 10^5^ L). (D) After 1 × 10^–2^ mbar for 1 min (4.5 × 10^5^ L). (E) After 1 × 10^–2^ mbar for 5 min (2.25 × 10^6^ L). (F) After 1 mbar for 10 min (4.5 × 10^8^ L).

**Figure 3:**
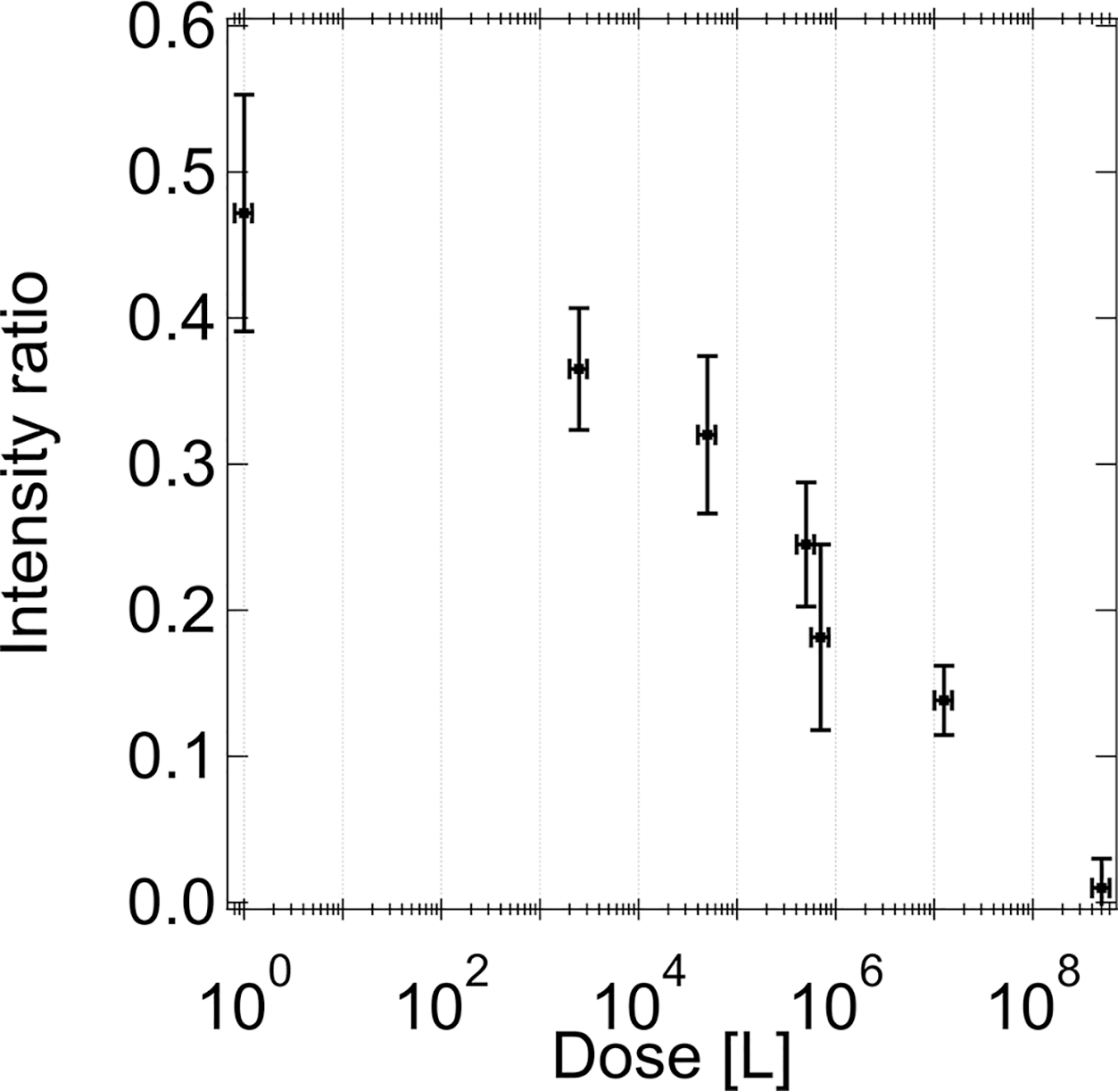
Ratio of the (2 × 1) reconstruction LEED spot intensity and the base (1 × 1) structure of TiO_2_(110) plotted vs the logarithm of the dose of water vapor. Quantification was obtained by averaging the intensity ratios of the following reciprocal lattice vector pairs: [2, −^1^/_2_] to [2, −1], [−2, −^1^/_2_] to [−2, −1], and [−2, ^1^/_2_] to [−2, 1]. We assign a nominal exposure of 1 L for the case shown in Figure 2A due to the residual water present in the vacuum vessel.

These results suggest that adsorbed formate can be displaced by water molecules arriving at the surface from the gas phase. As the dose of water progressively increases, formate displacement continues until, eventually, none is left at the surface, as suggested by the XPS results shown in Figure 1. The pressures of water vapor used in these experiments are reported to correspond to an increasing coverage of molecular H_2_O over a hydroxylated TiO_2_(110) surface.^22^ The disappearance of the (2 × 1) spots in LEED (Figure 2F) per se does not implicate a complete removal of the formate from the surface because van der Waals and Coulomb repulsion forces between individual formates can lead to a disordered structure due to surface diffusion along the [001] direction.^16^

Our findings appear to contradict the generally accepted scenario of the strong adsorption of formate and other organic contaminants on the TiO_2_ surface, which causes a hydrophobic behavior. However, it should be noted that only water was dosed on a HCOO^–^-saturated TiO_2_(110) surface in the experiments presented here. In this situation, the adsorbed formic acid is the limiting component for the surface chemical equilibrium. The presence of low partial pressures of HCOOH in the gas phase, which is the case in ambient air,^10^ may push back the equilibrium condition to a fully saturated surface with adsorbed formate species.

The stability of formate and water co-adsorption has been assessed by DFT total energy calculations of a set of optimized structures with a variable number of co-adsorbed formate and water molecules. Formate is known to strongly bind to TiO_2_(110) surfaces, due to the formation of a bridged, bidentate structure.^7^ A water molecule, however, can form only one bond with a lattice titanium atom. Considering all three scenarios, a rutile surface fully covered with molecularly bound formic acid, one fully covered with bidentate formate–hydroxyl pairs, and one with water, as shown in Figure S1, the adsorption energies per molecule obtained with respect to a clean surface are −1.04, −1.58, and −1.05 eV, respectively. These results show that bidentate formate–hydroxyl pairs indeed bind more strongly than molecularly bound formic acid and water to TiO_2_(110). However, because formates form two bonds with the lattice atoms, one can conclude that the increase in energy per bond is larger in the case of water and molecularly bound formic acid than for formate.

Reported STM images of HCOO^–^-saturated TiO_2_(110) show coverages of <100%.^23^ Therefore, our reference model is represented by the bridged formate-covered (BFC) surface with seven formates adsorbed on a 3 × 6 slab. This corresponds to a surface coverage of approximately 80%, where each formate bridges two neighboring titanium sites, as seen in Figure S2A. Such an arrangement leaves available adsorption sites that can be easily occupied by water molecules added *a posteriori*, as shown in Figure S2B–E. Also, by adding water as co-adsorbed species at the few remaining surface sites, we could confirm that formate binds more strongly, and increasing the coverage there affords a slight decrease in the adsorption strength, explained by the oversaturated surface. The results of these preliminary static calculations in a dry environment suggest that the structural changes observed by LEED and the loss of formate coverage seen by XPS must be due to thermally activated structural rearrangements. To identify the atomistic mechanisms that lead to these rearrangements, we carried out a series of equilibrium Born–Oppenheimer molecular dynamics (BOMD) simulations and additional enhanced sampling by metadynamics (MTD). For this scope, we selected different initial configurations and different MTD settings to explore more possible pathways that could explain the experimentally observed behavior.

The BOMD simulations in the canonical ensemble have been employed to monitor the equilibration of the adsorbates at finite temperature, which might already lead to local rearrangements. Two distinct surface structures 1 and 2 were considered, containing a different number of bidentate and monodentate radicals and co-adsorbed water molecules, as shown in panels A and B of Figure S3. Structure 1 contains seven bridged, bidentate formates and four co-adsorbed water molecules; structure 2 has three bridged, bidentate formates, four monodentate formates, four co-adsorbed water molecules hydrogen bonded to the monodentate radicals, and four water molecules co-adsorbed to the free titanium sites. Within the short time of the initial equilibration simulations, there is no evidence of spontaneous rearrangements that could be responsible for the experimentally observed modifications of the surface superstructure. This suggests that if reaction processes are going to occur, they would take place over longer time scales, have higher activation barriers, and possibly involve additional water molecules. Indeed, the few co-adsorbed water molecules are very stable and are not expected to induce the desorption of the neighboring bidentate and monodentate formates.

To better reproduce the experimental conditions, and increase the probability of structural rearrangements, structures 1 and 2 have been further equilibrated (10 ps at 300 K) after the addition of 11 and 9 extra water molecules, respectively. The additional molecules have been placed just above the first layer of adsorbates to saturate the hydrogen bonds, as shown in panels C and D of Figure S3. The further equilibration has not produced major changes in the arrangement of the co-adsorbates, but for some adjustments in the H-bond network, which in structure 2 induced the 90° rotation of one monodentate formate, similar to the case recently reported on the anatase (101) surface.^24^ This is a first indication that the interaction of the free oxygen atom of the formate with the surrounding water molecules might be associated with mobility and rearrangement processes. Our hypothesis is that the restructuring could occur stepwise, in which the first step is the transition from bidentate to monodentate. This would favor the interaction with neighboring water molecules, which in turn might trigger the further mobility of the adsorbates and formate–water exchange at some adsorption sites.

With this picture in mind, we decided to proceed with MTD simulations starting from different initial conditions and settings to explore different possible phases of the restructuring processes. The choice of collective variables in MTD allows for specifically targeting a foreseen process by biasing only a few degrees of freedom, thus selecting the desired pathway. Gaining more information from the sampling of the individual steps turns out to be helpful for observing the structural rearrangement under different conditions. Finally, we generalize the choice of the collective variable in an attempt to simulate the mechanism without an overly strong preselection for the involved molecules. The more general approach is expected to give a better estimate of the underlying free energy landscape. In the Supporting Information, we report the details of some preliminary simulations performed by activating the formate–water exchange for a specific pair, starting from structure 2, i.e., from an already monodentate formate.

In the case of the enhanced sampling performed by making use of structure 1, the description of the system lies between a more specific and a more general bias, including only one bridged, bidentate formate and all of the water molecules. In this simulation, MTD-1, four steps are observed for the substitution of the formate, with the final two being already observed during the other three simulations. First, surrounding water molecules interact with the formate, disturbing its configuration and changing it from bidentate to monodentate (step I to II, with a barrier Δ*G*_I,II_ of 0.24 eV), with subsequent adsorption of one water molecule onto the freed titanium site (step II to III, with a barrier Δ*G*_II,III_ of 0.06 eV), as shown in Figure 4B. Formation of H_3_O^+^, shown in panel C, takes place due to the interaction of a water molecule with a bridging hydroxyl. The hydronium ion then interacts with the formate, where a proton hops over to form a formic acid molecule. These proton transfers between different molecules are not directly addressed by our choice of CVs. They are, however, an indirect consequence of the water molecules approaching the surface and the different species present in the system. This process is then followed by the desorption of the formic acid molecule (step III to IV, with a barrier Δ*G*_III,IV_ of 0.38 eV), with subsequent adsorption of a second water molecule onto the freed site at the surface (step IV to V, with a barrier Δ*G*_IV,V_ of 0.10 eV), as shown in panels D and E of Figure 4.

**Figure 4:**
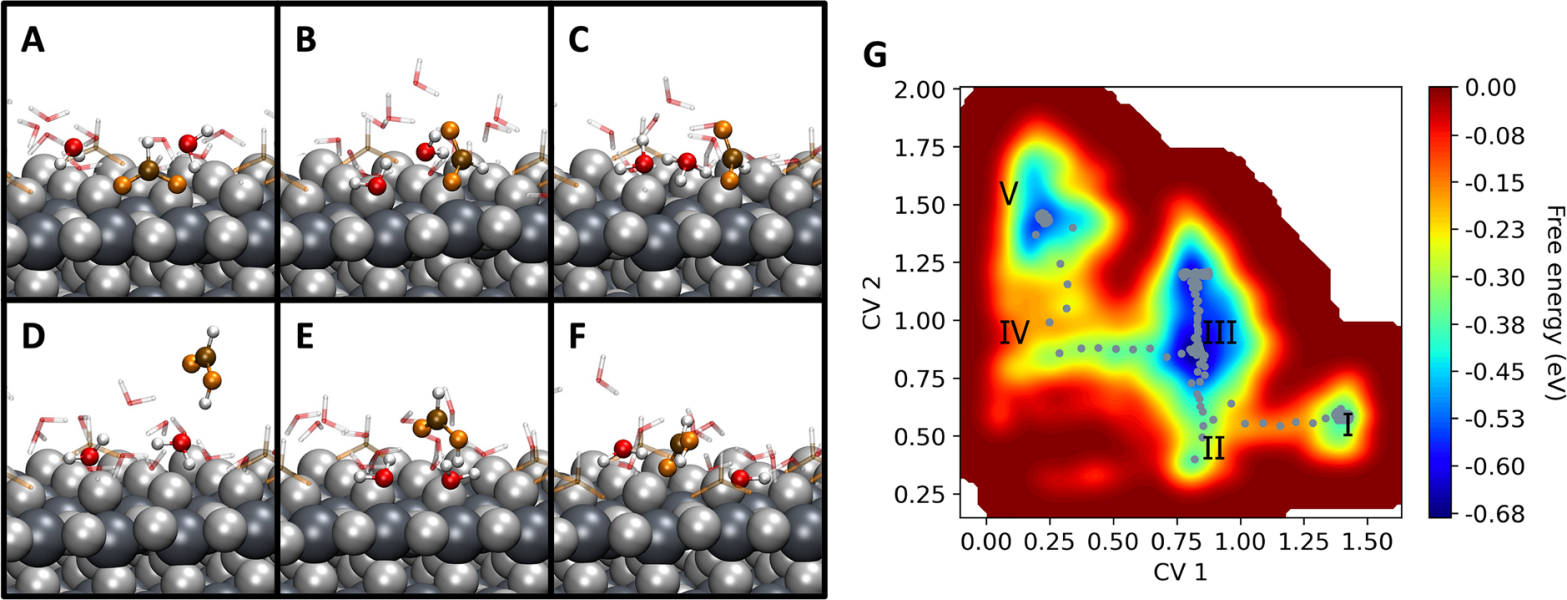
(A–F) Snapshots of the MTD-1 simulation. The lattice oxygen and titanium atoms are depicted as light and dark gray van der Waals spheres, respectively. The relevant molecules involved in the dynamic equilibrium are shown as balls and sticks, with oxygen atoms from water molecules colored red, carbon atoms colored brown, oxygen atoms from formate colored orange, and hydrogen atoms colored white. (A) Initial configuration employed in the MTD-1 simulation. (B) Formate changes its configuration from bidentate to monodentate, and the first water molecule gets adsorbed onto the surface. (C) Formation of the H_3_O^+^ ion from interaction of a water molecule with a bridging hydroxyl. (D and E) Formate desorbs as formic acid, and a second water molecule is adsorbed onto the freed titanium site. (F) Dynamic equilibrium between the water molecule and formic acid. (G) Free energy surface reconstructed from the Gaussian hills deposited during the simulation and minimum energy path shown as gray dots. CV1 is the collective variable (CV) representing the coordination number [CN (see the Supporting Information for a definition)] between two oxygen atoms of one adsorbed formate and the two titanium atoms of the adsorption site. CV2 represents the CN between all oxygen atoms from water molecules and the same two titanium atoms as for CV1. The relevant regions I–V are described in the text.

The free energy landscape estimated by our simulation shows barriers of few tenths of an electronvolt, consistent with a picture in which the room-temperature fluctuations of the hydrogen bond network^25^ might favor the exchange of protons and the rearrangement of the adsorbates. Because after desorption the formic acid molecule remains in the vicinity of the surface in our simulation, we also observe the reverse process, in which the molecule keeps approaching the surface, trying to remove the water molecule and bind again to the titanium sites, as shown in Figure 4 and Figure S4. This dynamic equilibrium process takes places until all simulations are converged, with the formate being re-adsorbed onto the surface always in the monodentate manner, which indeed turns out to be the configuration with the lowest free energy under the conditions of our model.

These combined experimental and theoretical results point toward a competitive adsorption mechanism for water molecules and formate on the surface. The ordered layer is disrupted as more water molecules can displace the previous adsorbates via dissociative adsorption and protonation of the formate. A series of static simulations considering different arrangements of formates and water molecules were performed. From the static calculations, however, it is not apparent how water co-adsorption can perturb the surface superstructure formed by the formate, suggesting it is necessary to expose the HCOOH-covered surface to extra water to allow the displacement of formate to occur. Metadynamics simulations considering different initial states and collective variables support the experimental findings. The calculations suggest a reaction path that takes place in four steps. The formate must desorb as formic acid, passing through an intermediate monodentate configuration, with adsorption of a first water molecule to a freed titanium site. Later, the monodentate formate desorbs as formic acid, leaving space for a second water molecule to bind to the lattice titanium atom. The energy barriers in the same order of magnitude as the H-bond strength at room temperature show that these processes can happen spontaneously through fluctuations of the H-bond environment. These results do not appear to fully agree with those of ref (10), in which contact with water in air still showed a (2 × 1) surface reconstruction. However, in that publication, no XPS or STM imaging was explicitly performed on an air-exposed or HCOOH-saturated surface that was subsequently exposed to water vapor in vacuum. While the observation of the (2 × 1) structure transforming into the (1 × 1) structure following exposure to water would appear to contrast well-established results,^2^ in our study only pure water was dosed over a TiO_2_(110) surface that was previously saturated with HCOOH. In experiments involving air exposure, a constant supply of formate is available in the form of a low partial pressure of formic acid.^23,25^ Ultimately, these results can contribute to the understanding of molecular processes happening during the adsorption of molecules on surfaces. Specifically, even in the case of strong adsorption, the chemical equilibrium between molecules present at the interface cannot be neglected.

## Data Availability

All data are available in the manuscript or the Supporting Information. Experimental and computational data are available for download under DOI: 10.5281/zenodo.7188707.

## Supporting Information Available

Movie generated from the MTD-1 trajectory, considering one of every 100 snapshots (Movie S1) (MP4)
Materials and Methods; top view of clean, water-saturated, dissociatively bound formic acid-saturated and molecularly bound formic acid surfaces (Figure S1); top view of the 80% bridged formate-covered surface with intermediate steps of water co-adsorption, up to a maximum of four molecules (Figure S2); top view of structures 1 and 2 before and after the addition of extra water molecules (Figure S3); snapshots of the MTD-2 simulation and reconstructed free energy surface (Figure S4); references 23–35; and parameters used in the metadynamics simulations (Table S1) (PDF)
Transparent Peer Review report available (PDF)
