# Supplementary material for: Dynamic Equilibrium at the HCOOH-Saturated TiO2(110)–Water Interface
Source: J Phys Chem Lett. 2023 Mar 23;14(13):3132–8. doi: 10.1021/acs.jpclett.2c03788 (PMC10084457; doi:10.1021/acs.jpclett.2c03788)
Supplement: Supplementary file 2 — jz2c03788_si_002.pdf [file jz2c03788_si_002.pdf]

Supplementary information

Dynamic Equilibrium at the HCOOH-saturated TiO_2(110)-Water Interface

Fernanda Brandalise Nunes[1*], Nicolò Comini[2,3*], J. Trey Diulus[2,3], Thomas Huthwelker[3], Marcella Iannuzzi[1]‡, Jürg Osterwalder[2], Zbynek Novotny[2,3,4]‡

[1] Department of Chemistry, University of Zürich, CH-8057 Zürich, Switzerland
[2] Department of Physics, University of Zürich, CH-8057 Zürich, Switzerland
[3] Swiss Light Source, Paul Scherrer Institut, CH-5232 Villigen-PSI, Switzerland
[4] Empa, Swiss Federal Laboratories for Materials Science and Technology, Laboratory for Joining Technologies and Corrosion, CH-8600 Dübendorf, Switzerland

*These authors contributed equally to this work
‡Corresponding author. Email: marcella.iannuzzi@chem.uzh.ch, zbynek.novotny@empa.ch

This PDF file includes:

Materials and Methods
Supplementary Text
Figs. S1 to S4
Table S1

Other Supplementary Materials for this manuscript include the following:

Movie S1

Materials and Methods

Experimental details. The experiments were performed using the Solid-Liquid Interface Chamber (SLIC) endstation at the Swiss Light Source of the Paul Scherrer Institute, described in detail elsewhere.[1] XPS spectra were acquired at the PHOENIX I beamline using a 4 keV photon beam, while low-energy electron diffraction (LEED) experiments under variable water dosing were performed in the endstation as a standalone system. XPS measurements were performed on a rutile $TiO_2(110)$ single crystal (floating zone material, one-side polished) with dimensions of 50×10×1 mm^3 (SurfaceNet GmbH). This sample was prepared by cycles of Ar^+ sputtering and vacuum annealing using the custom-made oven in a separate chamber described in Refs. 1, 2. The chamber where the annealing took place had a base pressure of 10^{-8} mbar during the experiments, while it was $1x10^{-10}$ mbar in the preparation/LEED chamber. For the LEED measurements shown in Fig. 2, a smaller 7×7×0.5 mm^3 sample (PI-KEM Ltd.) of the same material was used, prepared by Ar^+ sputtering and annealing via electron beam heating. The exposure to water vapor during APXPS experiments was performed in a non-baked chamber with a base pressure in the 10^{-9} mbar range. Conditions for XPS measurements correspond to those described in Ref. 2.

LEED images were acquired using a very low incident electron beam current of nominally 40 nA to minimize any possible electron-induced processes.[3] Using 100 eV electron energy, the diffraction signal was multiplied by a pair of microchannel plates (MCPs) with an 800 V potential applied. The sample was irradiated with electrons only during LEED acquisition (4 seconds) when a mechanical shutter was opened. Test measurements were performed, rendering the influence of electron-stimulated desorption negligible due to the electron beam flux being ~50× lower than conventional LEED instruments. Figures shown in the main text have been corrected for the radial distortion due to the planar channel-plate detector and treated to remove background signals and enhance the contrast corresponding to the underlying (1×1) surface structure and the (2×1) reconstruction formed following the exposure to HCOOH. Quantification of LEED intensities was performed on the raw images by using an STM package plugin for ImageJ.[4] The analysis consisted of background removal, followed by numerical integration of the peak intensity.

MilliQ Type 1 water was used in all experiments. For APXPS experiments the water was outgassed using a single freeze-pump-thaw cycle in a dedicated vacuum-sealed desiccator, before inserting it into the analysis chamber via a short exposure to air.[1] Sample was dipped into liquid water using the dip-and-pull technique (for more details, see Refs. 1, 2). For LEED measurements, a water reservoir was attached to the analysis chamber via a high-precision leak valve. Prior to the experiments, it was purified by four freeze-pump-thaw cycles using liquid nitrogen for freezing, followed by pumping with a turbomolecular pump. The same procedure was applied to liquid formic acid (Merck, 98-100%, EMSURE® ACS, Reag. Ph Eur), which was then dosed through a different high-precision leak valve. The reported doses do not account for the time required to reach the exposure pressure. The nominal exposure doses reported in Fig. 2 are therefore rounded upwards to account for the transient phase. Due to the higher relative transient dose in performing the experiment shown in Fig. 2D and 2E, the dose is respectively corrected to a nominal 7×10^5 L and 4×10^6 L.

Ab initio Simulations. In order to study the competitive adsorption between formate and water on the rutile surface, Kohn-Sham density functional theory (KS-DFT)[5, 6] simulations have been carried out employing the Perdew–Burke-Ernzerhof (PBE)[7] exchange-correlation functional

supplemented with the revised version of the nonlocal density functional by Vydrov and Van Voorhis (rVV10).[8] The simulations have been performed using the CP2K program,[9] describing the valence electrons only, under the Gaussian and plane-wave (GPW) approach. GPW needs to be associated to the use of pseudopotentials to account for the interactions of valence electrons with the atomic core. We employed norm-conserving GTH pseudopotentials,[10] triple-zeta valence plus polarization (TZV2P) basis sets, and a cutoff of 600 Ry for the auxiliary plane-wave basis set. This approximation is accurate for standard ground state electronic structure calculations and as driver for molecular dynamics (MD) simulations.

The simulations are performed with a $TiO_2(110)$ slab model consisting of four trilayers of 3×6 units, corresponding to a simulation cell of 19.66×17.81×33.11 Å^3 (including 20 Å vacuum in the z-direction). Periodic boundary conditions have been applied for each simulation. The exposed $TiO_2(110)$ surface in our model offers nine sites for the adsorption of formic acid, which binds dissociatively, in a bridged, bidentate configuration.[11] Each site consists of two neighboring titanium atoms. Water adsorption takes place molecularly, with one water molecule per titanium site, i.e., eighteen adsorption sites on the bare slab. As reference configuration, we consider the 80% bridged-formate-covered (BFC) surface, thus leaving four bare titanium sites, as shown in panel A of Fig. S2. Water adsorption energies are then obtained with respect to the 80% BFC surface, i.e.,

$$\mathrm{E}^{\mathrm{ADS}} = \mathrm{E}_{80\%}^{\mathrm{n\,H_2O}} - \mathrm{E}_{\mathrm{BFC}} - \mathrm{n}\,\mathrm{E}_{\mathrm{H_2O}},$$

where $E_{80\%}^{n\,H_2O}$ is the energy of the whole system, consisting of 80% covered surface and n water molecules, E_{BFC} is the energy of the reference system, E_{H2O} is the energy of one water molecule in gas-phase.

The Born-Oppenheimer molecular dynamics (BOMD) simulations are carried out in the canonical ensemble (NVT). Two coverages have been selected for the finite temperature equilibration, and their surface structures are shown respectively in the panels A and B of Figure S3:

1. Seven bridged bidentate formates plus four water molecules.
2. Three bridged bidentate formates, four monodentate formates, four co-adsorbed water molecules hydrogen bonded to the monodentate radicals, and four co-adsorbed water molecules on the empty titanium sites.

The initial equilibration is obtained by a series of three short MD runs (5 ps each) at increasing temperature (100, 200, and 300 K). During these simulations the pre-selected configurations turn out to be stable, since no important rearrangements were observed, such as reaction or substitution of the adsorbed formate groups by water molecules. To better reproduce the experimental conditions, and increase the probability of structural rearrangements, structures 1 and 2 have been further equilibrated (10 ps at 300 K) after adding 11 and 9 extra water molecules, respectively, just above the first layer of adsorbates to saturate the hydrogen bonds, as shown in panels C and D of Figure S3.

To enhance the sampling of the configurations space, we applied the metadynamics (MTD)[12] approach on top of BOMD. MTD is a successful technique that allows the simulation of processes

by selecting a few relevant collective variables (CVs, $(s_1, s_2, \ldots, s_{n_s})$) that optimally describe the aimed process. By MTD the sampling in the reduced space of the selected CVs is accelerated by means of the time-dependent MTD potential, which is built on the fly as a sum of sequentially spawned Gaussian contributions. The time interval between two consecutively added contributions (τ_G), as well as the size of the Gaussians (height W and width σ_i), are system-dependent parameters.

For the simulations presented in this work, we opted for CVs representing generalized coordination numbers (CN). This choice allows for control of the coordination environment of selected atoms or species without too strongly biasing towards pre-determined structures. The CN is expressed as a function of the atomic coordinates of pre-selected lists of atoms as

$$CN_{A_1,A_2} = \frac{1}{N_{A1}} \sum_{i_{A_1}} \sum_{j_{A_2}} \frac{1-(r_{ij}/R_o)^{NN}}{1-(r_{ij}/R_o)^{ND}},$$

where A_1 and A_2 are the two sets of atoms for which the CN is computed, r_{ij} is the distance between two atoms, one belonging to set A1 and the other to set A2. The reference distance R_o and the exponents *NN* and *ND* determine the extension of the coordination range.

We did four MTD runs, the first starting from structure 1 and the other three starting from structure 2, in both cases after the addition of the extra water layer. Each MTD run is carried out with two CN as CVs, as described in the following.
MTD-1: for CV1, the A1 set contains two oxygen atoms of one adsorbed formate and A2 contains two titanium atoms of the adsorption site. For CV2, A1 contains all oxygen atoms from water and for A2, the same two titanium atoms as for CV1.
MTD-2: for CV1, the A1 set is only one O atom of the rotated formate bound to the surface, the A2 set is the titanium atom of the corresponding adsorption site; for CV2 the A1 set is the same titanium atom and the A2 set contains the oxygen atom of one water molecule in the overlayer. For this simulation the excursion of CV1 is limited by an external potential that prevents the desorbing formic acid to move too far from the surface. Such a strategy is consistent with the assumption that the desorbed formic acid can be replaced by other equivalent molecules which are always present in the gas phase above the surface.
MTD-3: for CV1, the A1 set contains all formates' oxygen atoms bonded to the surface, i.e., two from each bridged, bidentate formate, and one from each monodentate radical. Set A2 contains all the corresponding surface titanium atoms. For CV2, the A1 set contains all waters' oxygen atoms and set A2 all the surface titanium atoms of the formates' adsorption sites.
MTD-4: for CV1, the A1 contains all formates' oxygen atoms which are bonded to the surface, and A2 contains the Ti atoms of the corresponding adsorption sites. For CV2 the A1 set contains all formates' oxygen atoms (two from each formate) and A2 all waters' hydrogen atoms.

In all four MTD simulations, Gaussians with a height of 0.5×10^{-3} Ha were deposited every 30 fs, with $NN = 8$ and $ND = 12$. The four MTDs have been extended until the free energy surface (FES) reconstructed from the MTD potential have been considered converged. The total simulation times and the relevant parameters for the CN functions are reported in Table S1.

Supplementary Text

Additional static DFT-based simulations

Intermediate steps of water co-adsorption have been obtained by adding one water molecule at a time, close to one of the remaining available adsorption sites, up to a maximum of 4 molecules, to the BFC structure. Each new configuration, shown in panels B-E of Fig. S2, have been optimized and the gain in energy has been estimated by calculating the adsorption energy of the additional water molecule. For all four molecules, the co-adsorption process showed to be favorable, with the largest energy gain, of -1.05 eV, obtained after adding the first water molecule, and the smallest, of -0.81 eV, after adding the third one. The differences in adsorption energy at the four different sites could be explained by the surrounding environment. For the first two water molecules, there were still empty neighboring sites close to the chosen adsorption sites. Consequently, the gain in energy in these two cases were the largest ones. For the last two molecules, all the neighboring sites were occupied by either formates or water molecules, and the gain in energy was consequently smaller.

Infrared spectroscopy studies on P25 TiO_2[13] showed that exposure to water appears to perturb the structure of adsorbed formate, changing its conformation from bidentate to monodentate, a more reactive configuration. For this reason, we also considered in our simulations formate groups adsorbed as a monodentate. A combined surface shown in panel B of Figure S3, including monodentate formates, was also built, containing three formate groups adsorbed in a bridged, bidentate configuration, and four adsorbed in a monodentate manner. There are also eight water molecules co-adsorbed at the surface, four to neighboring sites of the monodentate formate, and four to the remainder empty titanium sites.

Additional MTD simulations

As mentioned in the main text, we started our simulations from a more specific bias of a few degrees of freedom. We first focused on the process starting from a specific formate already in the monodentate configuration, biasing towards the precise exchange of this formate with one neighboring water molecule. This simulation, MTD-2, shows that the substitution of the formate with water molecules happens in two distinct steps. The biased formate is already rotated by 90º degrees, with the free oxygen atom interacting with a bridging hydroxyl group of the surface, forming a formic acid molecule. The interaction of the formed molecule with surrounding water molecules pushes the formic acid away from its original adsorption site, making the molecule to desorb from the surface. In a second step, one of the surrounding water molecules approaches and binds to the freed titanium site, with the complete replacement of the formate with water molecules.

Going towards a more general description of the system, all formates and all water molecules were considered, either by selecting the oxygen (MTD-3) or the hydrogen atoms (MTD-4) of the water molecules. In these simulations, we also observed the desorption of the formate that leaves the surface in MTD-2. In MTD-4, desorption of a second monodentate formate was also observed. The hydrogen atom that binds to formate to create the formic acid molecule, in this case, comes from a neighboring water molecule (rather than from a bridging hydroxyl), and the remaining hydroxyl group binds to the freed titanium atom. The substitution steps observed during MTD-2 were also followed during the desorption process in simulations MTD-3 and MTD-4.

Among all the MTD simulations performed, only MTD-1 and MTD-2 can energetically describe the processes taking place at the surface. This is the case due to the choice of CVs for MTD-3 and MTD-4. Since many atoms are involved in the CV description, it is not straightforward to assign the process taking place at the titanium dioxide surface to specific regions of the FES. The FES obtained from the MTD-2 simulation is shown in panel G of Fig. S4, together with snapshots of the trajectory (panels A-F). In the case of MTD-2, in which a monodentate formate is being considered in the CV description, we can assign the following processes:

I to II. formate desorbs as formic acid, with barrier $\Delta G_{I,II} = 0.42$ eV;

II to III. water molecule gets adsorbed to the surface, with barrier $\Delta G_{II,III} = 0.18$ eV.

Even though the surface structure and the collective variables considered in MTD-1 and MTD-2 simulations are different, it is possible to have a good agreement between both cases, showing that the choice of collective variables for this problem was appropriate.

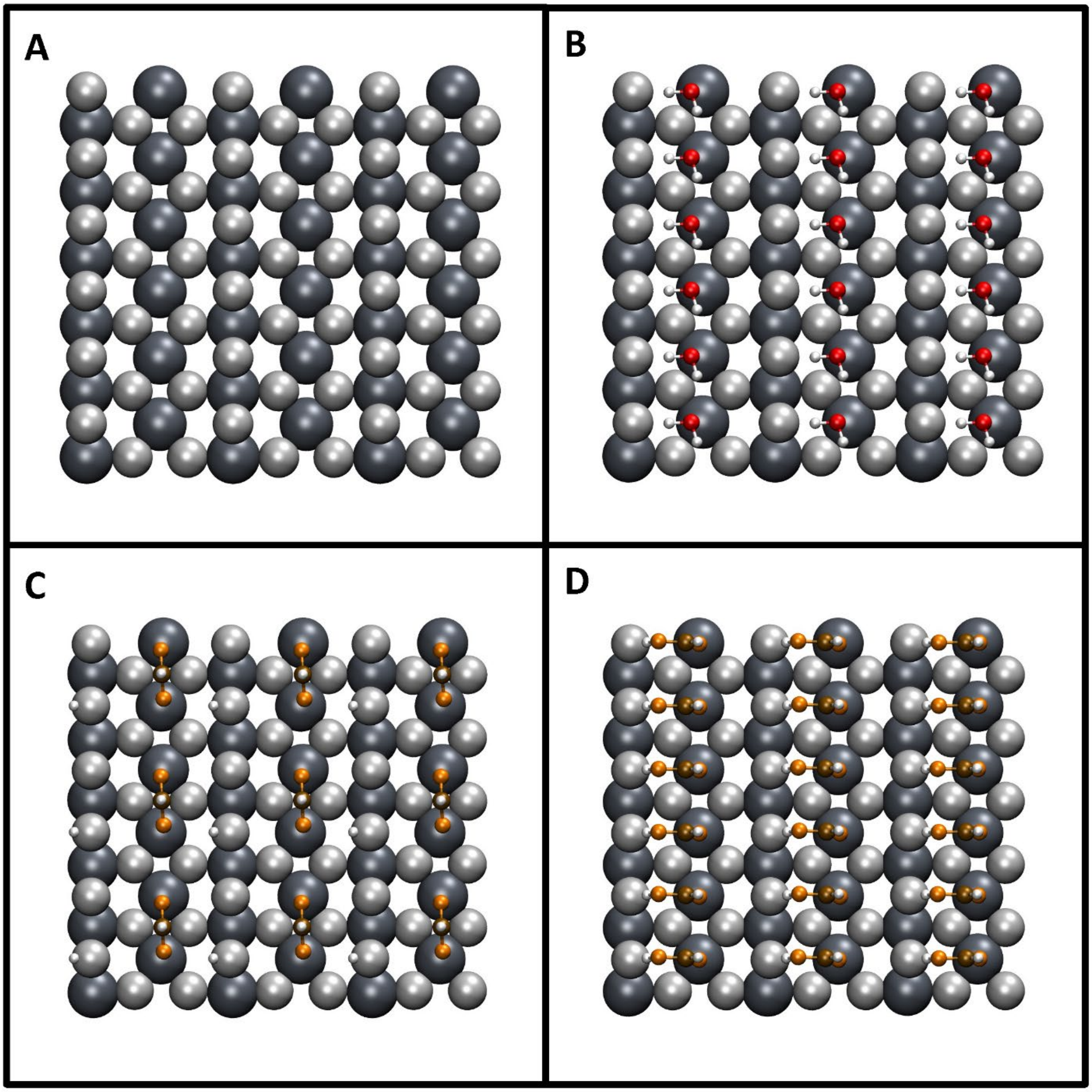

Fig. S1.

Top view of (A) clean, (B) water-saturated (E^{ADS} = -1.05 eV per water molecule), (C) dissociatively-bound formic acid-saturated (E^{ADS} = -1.58 eV per bidentate formate-hydroxyl pair), and (D) molecularly-bound formic acid-saturated (E^{ADS} = -1.04 eV per formic acid molecule) $TiO_2(110)$ structures. Lattice atoms are depicted as van der Waals spheres, with oxygens in light and titanium in dark grey. Water molecules, formates and hydrogen atoms are in balls and sticks representation. Oxygen atoms from water molecules are shown in red, carbon atoms are shown in brown, formate and formic acid oxygens in orange, and hydrogen atoms in white. Only the surface atoms are shown in all panels.

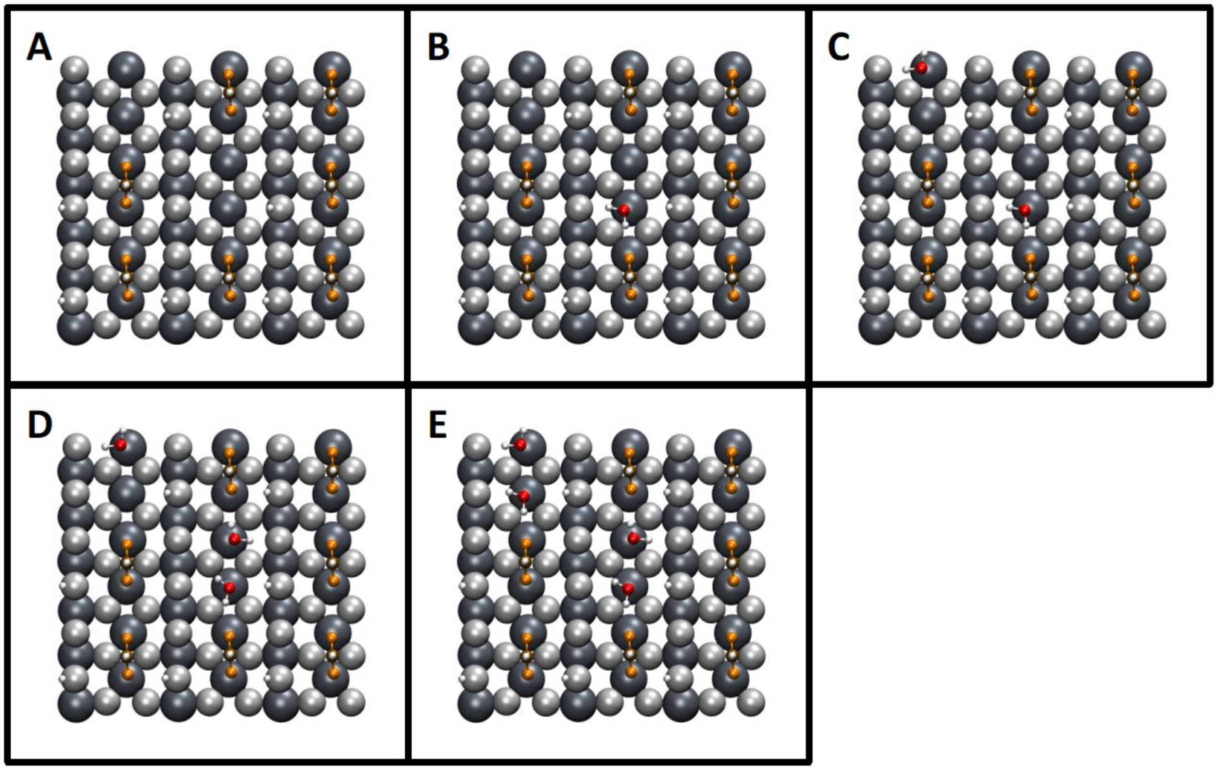

Fig. S2.

Top view of the 80% bridged-formate-covered surface with intermediate steps of water co-adsorption, up to a maximum of 4 molecules. (A) Optimized BFC, with an average adsorption energy of - eV per bidentate formate-hydroxyl pair with respect to the clean surface. BFC with (B) one, (C) two, (D) three, and (E) four co-adsorbed water molecules. The water molecules' adsorption energies were calculated with respect to the previous step (i.e., structure with one water molecule less), and have values -1.05, -0.93, -0.81, and -0.92 eV, for structures depicted in panels (B)-(E) respectively. Only the surface atoms are shown in all panels.

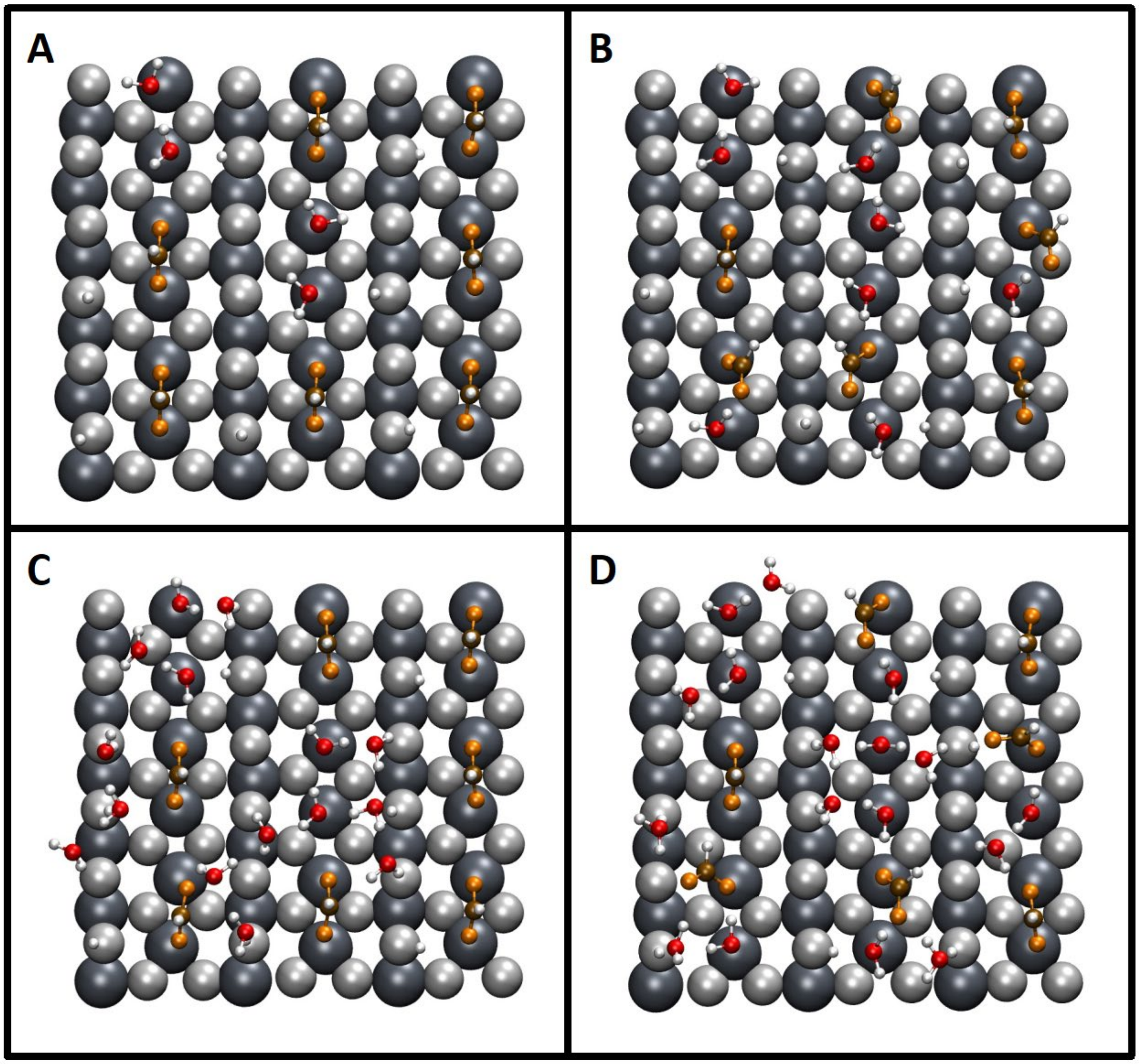

Fig. S3.

Top view of structures 1 and 2 before and after the addition of extra water molecules. (A) Equilibrated structure 1 before addition of extra water molecules. (B) Equilibrated structure 2 before addition of extra water molecules. (C) Equilibrated structure 1 after addition of extra 11 water molecules. (D) Equilibrated structure 2 after addition of extra 9 water molecules. Lattice atoms are depicted as van der Waals spheres, with oxygens in light and titanium in dark grey. Water molecules, formates and hydrogen atoms are in balls and sticks representation. Oxygen atoms from water molecules are shown in red, carbon atoms are shown in brown, formate oxygens in orange, and hydrogen atoms in white. Only the surface atoms are shown in all panels.

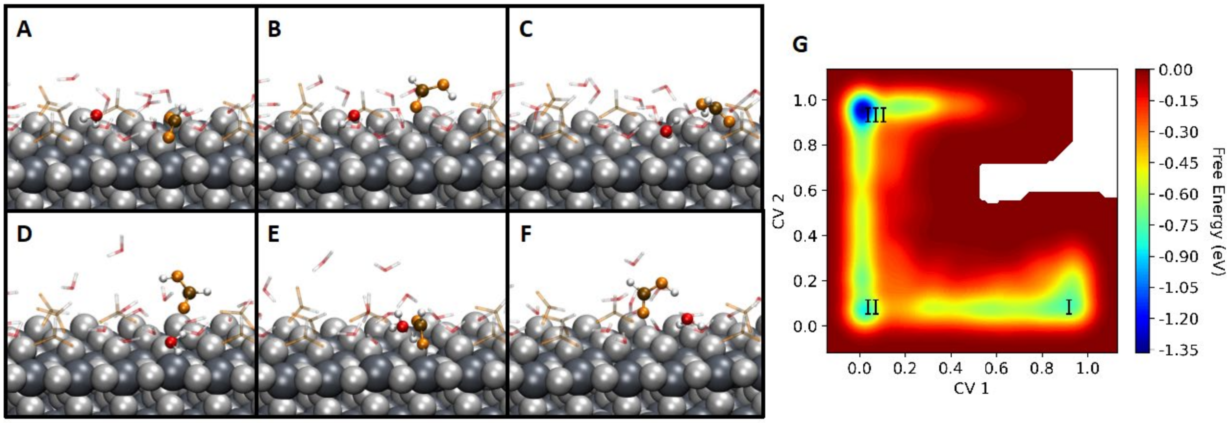

Fig. S4.

(A-F) Snapshots of MTD-2 simulation. The relevant molecules involved in the dynamic equilibrium are shown as balls and sticks. (A) Initial configuration employed in MTD-2 simulation. (B) Formate desorbs as formic acid. (C) Water molecule is adsorbed onto the freed titanium site. (D-F) Dynamic equilibrium between water molecule and formic acid. (G) Free energy surface reconstructed from the Gaussian hills deposited during the simulation. The relevant regions I-III are described in the text.

Table S1.
Total simulation times and relevant parameters for the calculation of the coordination number functions of all four metadynamics simulations.

Simulation	Simulation time (ps)	CV	R_o (Å)	Width (in units of CN)
MTD-1	48.5	1	2.9	0.05
		2	2.9	0.05
MTD-2	34.5	1	2.9	0.05
		2	2.9	0.05
MTD-3	31.6	1	2.9	0.05
		2	2.9	0.03
MTD-4	31.5	1	2.9	0.05
		2	1.6	0.05

References

(1) Novotny, Z.; Aegerter, D.; Comini, N.; Tobler, B.; Artiglia, L.; Maier, U.; Moehl, T.; Fabbri, E.; Huthwelker, T.; Schmidt, T. J.; et al. Probing the solid–liquid interface with tender x rays: A new ambient-pressure x-ray photoelectron spectroscopy endstation at the Swiss Light Source. *Rev. Sci. Instrum.* **2020**, *91* (2), 023103 (acccessed 2020/02/14).
(2) Comini, N.; Huthwelker, T.; Diulus, J. T.; Osterwalder, J.; Novotny, Z. Factors influencing surface carbon contamination in ambient-pressure x-ray photoelectron spectroscopy experiments. *J. Vac. Sci. Technol. A* **2021**, *39* (4), 043203 (acccessed 2021/05/20).
(3) Chambers, S. A.; Thevuthasan, S.; Kim, Y. J.; Herman, G. S.; Wang, Z.; Tober, E.; Ynzunza, R.; Morais, J.; Peden, C. H. F.; Ferris, K.; et al. Chemisorption geometry of formate on TiO_2(110) by photoelectron diffraction. *Chem. Phys. Lett.* **1997**, *267* (1), 51-57.
(4) Choi, J. I. J.; Mayr-Schmölzer, W.; Mittendorfer, F.; Redinger, J.; Diebold, U.; Schmid, M. The growth of ultra-thin zirconia films on Pd_3Zr(0001). *J. Phys.: Condens. Matter* **2014**, *26* (22), 225003.
(5) Hohenberg, P.; Kohn, W. Inhomogeneous electron gas. *Phys. Rev.* **1964**, *136* (3B), B864-B871.
(6) Kohn, W.; Sham, L. J. Self-consistent equations including exchange and correlation effects. *Phys. Rev.* **1965**, *140* (4A), A1133-A1138.
(7) Perdew, J. P.; Burke, K.; Ernzerhof, M. Generalized gradient approximation made simple. *Phys. Rev. Lett.* **1996**, *77* (18), 3865-3868.
(8) Sabatini, R.; Gorni, T.; de Gironcoli, S. Nonlocal van der Waals density functional made simple and efficient. *Phys. Rev. B* **2013**, *87* (4), 041108.
(9) Kühne, T. D.; Iannuzzi, M.; Ben, M. D.; Rybkin, V. V.; Seewald, P.; Stein, F.; Laino, T.; Khaliullin, R. Z.; Schütt, O.; Schiffmann, F.; et al. CP2K: An electronic structure and molecular dynamics software package - Quickstep: Efficient and accurate electronic structure calculations. *J. Chem. Phys.* **2020**, *152* (19), 194103.
(10) Goedecker, S.; Teter, M.; Hutter, J. Separable dual-space Gaussian pseudopotentials. *Phys. Rev. B* **1996**, *54* (3), 1703-1710.
(11) Diebold, U. The surface science of titanium dioxide. *Surf. Sci. Rep.* **2003**, *48* (5–8), 53-229.
(12) Laio, A.; Parrinello, M. Escaping free-energy minima. *Proc. Natl. Acad. Sci. USA* **2002**, *99* (20), 12562-12566 (acccessed 2022/07/25).
(13) Miller, K. L.; Lee, C. W.; Falconer, J. L.; Medlin, J. W. Effect of water on formic acid photocatalytic decomposition on TiO_2 and Pt/TiO_2. *J. Catal.* **2010**, *275* (2), 294-299.
