# Supplementary material for: Dynamic Equilibrium at the HCOOH-Saturated TiO2(110)–Water Interface
Source: J Phys Chem Lett. 2023 Mar 23;14(13):3132–8. doi: 10.1021/acs.jpclett.2c03788 (PMC10084457; doi:10.1021/acs.jpclett.2c03788)
Supplement: Supplementary file 3 — jz2c03788_si_003.pdf [file jz2c03788_si_003.pdf]

jz-2022-03788e.R1

Name: Peer Review Information for "Dynamic equilibrium at the HCOOH-saturated TiO_2(110)-water interface"

First Round of Reviewer Comments

Reviewer: 1

Comments to the Author

This manuscript reports a combined experimental (XPS, LEED) and computational study of the competitive adsorption of formic acid and water with rutile TiO2 (110) surface. The results suggest a dynamic equilibrium between formic acid and water adsorption, challenging the view that there is a selective high affinity adsorption of atmospheric formic and acetic acid on TiO2(110) which can explain the hydrophobic/ hydrophilic switching behavior of this surface under dark conditions/UV illumination (Ref. 9).

While I find these results potentially interesting, I believe they are still too preliminary to be convincing and publishable. This is particularly true for the computational results, that involve only a very limited exploration of configuration space in terms of both BOMD simulations (too short for equilibration, particularly for the large system used in this study) and MTD trajectories (only four, with very ad hoc initial configurations and CVs).

Similarly, also the analysis of the XPS spectra is very quick and qualitative, with no detailed discussion of the changes in the peaks and the kinetics, especially for the dominant peak around 285 eV.

An additional remark: the manuscript frequently mentions “formate radicals”. Such species do not exist!

Reviewer: 2

Comments to the Author

The authors present a new study on the dynamic equilibrium between water vapor and a rutile TiO2(110) surface saturated with a (2x1) overlayer of HCOOH. The authors present compelling evidence for the restructuring of a formate HCOO- monolayer on TiO2 by exposing a saturated surface with sequential dosing of water vapor.

I recommend publishing in J. Phys. Chem. Lett. with the following mandatory revisions listed below. I am also requesting to see the revised draft.

1. The dissociative chemisorption of formic acid on rutile TiO2(110) has been well-documented over the past 25 years. Throughout the manuscript, the authors refer to the chemisorbed formate anion (HCOO-) as a “formate radical”. This is incorrect. Formic acid dissociatively chemisorbs on the TiO2(110) as a

formate anion to undercoordinated Ti4+ and the proton on the bridging oxygen. The undercoordinated Ti4+ sites act as a Lewis Acid, and the bridging oxygen as a Lewis Base.

The article, as written, must be changed to reflect the correct anionic character of the formate species. Dissociative chemisorption of carboxylic acids on TiO2 is not a radical-based mechanism.

Related issues: In the abstract, the authors state that the bidentate binds “bidentate way”. This sounds rather strange; please reformulate and remove the word “way”. Also, on page 12, lines 21-23, the authors state that “The hydronium ion then interacts with the formate radical, where a hydrogen atom hops over to form a formic acid molecule.” Formally, it is a proton that is transferred, not hydrogen atoms. Please correct.

2. In the introduction, the authors write, “While in principle, different adsorption geometries are in principle possible…” Previous researchers have observed monodentate species.1

3. The adventitious carbon XPS peak is much larger than that of HCOO carbon. The relative coverages of both species should be quantified. The authors state that coverage of HCOO carbon is calculated to be ~0.5 ML. This is inconsistent with the large adventitious C seen in Figure 1. It is also surprising that such a large amount of the adventitious C would allow for the formation of the (2×1) phase in LEED. Please explain how such a large amount of adventitious C can coexist with the ordered (2×1) phase.

4. Figure 2 shows processed LEED images of saturated HCOOH-terminated surfaces after sequential exposure to H2O vapor. Please provide corresponding XPS C1s data from water exposures. Very little is discussed with respect to the relationship between the LEED changes and the chemistry that occurs on the surface. High-resolution scans of the C1s region would provide this information and increase the impact of this manuscript. The levels of adventitious C in such data should also be provided. Additionally, the time-dependent stability of the (2x1) overlayer under the x-ray and e- beams should be discussed.

5. Several published manuscripts show that water rinses self-assembled monolayers off.2 Please discuss the prior studies and the factors (kinetics, solvation) that are known to lead to washing off the self-assembled monolayers.

6. When describing Figure S1, the authors state on pg. 8 that panel (a) shows a rutile surface fully covered with formic acid. The description should state that the surface is covered by bidentate hydroxyl pairs. Additionally, please add another panel showing molecularly-bound formic acid and its energetics. This will complement the discussion and illustrate the relative binding energy of molecularly-bound water and formic acid species. Further, please include the per-molecule binding energies in the figure or the caption.

7. Figures S2 and S3 – include the per-molecule binding energies in the figure or the caption.

8. Page 9, paragraph 2 – list the temperature and water pressure used in the BOMD simulations.

9. In the discussion of Figure 4, it is not clear whether the listed values of the free energies refer to the exergonicity of the steps or to their barriers. Both values are important and should be listed for each elementary step. Further, free energies are usually indicated using the symbol G, not E, and relative values are indicated by using the symbol delta. Please list the values of free energies in panels A-F. The last panel in Figure 4 presents the 2-dimensional free energy diagram. For clarity and the benefit of the

readers, it would be very useful to graphically present the lowest free energy path along the reaction coordinate with the set of elementary steps.

10. Having the free energy dependencies should provide a straightforward path to construct the pressure and temperature-dependent kinetics of formate removal—something the authors could consider.

11. List the experimental temperature on page 4, line 2. On line 3, page 4, list the colors of the spectra that are being referred to in the text.

References

1. A. Mattsson, S. Hu, K. Hermansson, L Osterlund, "Adsorption of formic acid on rutile TiO2(110) revisited: An infrared reflection-adsorption spectroscopy and density functional theory study" J. Chem. Phys. 140, 034705 (2014).

2. E. Skibinski, W. J. I. DeBenedetti, and M. A. Hines, "Solution Deposition of Phenylphosphinic Acid Leads to Highly Ordered, Covalently Bound Monolayers on TiO2(110) Without Annealing" J. Phys. Chem. C 121 14213-14221 (2017).

Reviewer: 3

Comments to the Author

1. What is the major advance reported in the paper?

The study uses a combination of experimental and theoretical methods to examine the interaction of water with an adsorbed formate layer on rutile TiO2 (110). Photoemission measurements provide convincing evidence that dipping in water causes formate to be desorbed, with LEED results being consistent with this finding. The theoretical section details a reaction mechanism in which the water aids the desorption of the bound formate species.

2. What is the immediate significance of this advance?

This paper presents a challenge to a previous study (Balajka et al, Science 2018--ref 9) that concluded that formate binds preferentially over water (and other adsorbates) on rutile, creating a hydrophobic surface. The key difference highlighted is the effect of chemical equilibria on formate adsorption, such that the partial pressure of formic acid in air and/or solution concentration will play a key role in determining the nature of the contact layer. This is critical for an understanding in a number of applications, such as self-cleaning glass.

Given the major finding of the work, it important to correct an inaccuracy in the manuscript introduction regarding refs 8 and 9. Ref 8 concluded that OH was formed at the contact layer after dipping and immersing the (110) substrate in water. This is not accurately described as 'restructuring' as stated in the manuscript. Ref 9 re-interpreted these earlier results as formate in the contact layer based on the supposed displacement of water by formate, and the assumption that there was a small concentration of the acid in the ultra-pure water employed in the earlier work. The results of the current work rule out

the latter interpretation, which is a significant finding that should be brought out more in the discussion section.

3. Technical suggestions

The dipping procedure should be described in the current manuscript (SI).

A low current LEED apparatus was employed, but was a test carried out to ensure the absence of beam damage?

It is not clear how many water molecules were included in the MD simulations. Is it the number shown in the movie?

Author's Response to Peer Review Comments:

Please see attached Word document containing response to the reviewer's comments.

Reply to the editor: All non-scientific changes have been performed.

Reviewer(s)' Comments to Author:

Reviewer: 1

Recommendation: This paper is not recommended because the material is not appropriate for The Journal of Physical Chemistry Letters.

Comments:
This manuscript reports a combined experimental (XPS, LEED) and computational study of the competitive adsorption of formic acid and water with rutile TiO_2 (110) surface. The results suggest a dynamic equilibrium between formic acid and water adsorption, challenging the view that there is a selective high affinity adsorption of atmospheric formic and acetic acid on TiO_2(110) which can explain the hydrophobic/ hydrophilic switching behavior of this surface under dark conditions/UV illumination (Ref. 9).

While I find these results potentially interesting, I believe they are still too preliminary to be convincing and publishable. This is particularly true for the computational results, that involve only a very limited exploration of configuration space in terms of both BOMD simulations (too short for equilibration, particularly for the large system used in this study) and MTD trajectories (only four, with very ad hoc initial configurations and CVs).
Similarly, also the analysis of the XPS spectra is very quick and qualitative, with no detailed discussion of the changes in the peaks and the kinetics, especially for the dominant peak around 285 eV.

An additional remark: the manuscript frequently mentions "formate radicals". Such species do not exist!

Reply: We are sorry that Reviewer 1 does not agree with us about the importance and accuracy of the work presented.
We would like to remark that to prepare the model for the molecular dynamics simulations, we have carried out a preliminary study on the structure of the adsorbates' layer. We performed a series of structure optimizations starting from several different configurations and comparing different adsorption modes as well as different coverages, for formic acid, water and the two combined. The main results are reported in the supplementary information. Adsorption energies and projected density of states have been taken into account to select the optimal initial configurations to be employed in the dynamic simulations. The resulting initial configurations are not ad-hoc, they correspond to the experimentally observed superstructure, to which a small amount of water is added.
We would like to clarify that the BOMD simulations have been performed only for 5 ps at 100, 200, and 300 K to further equilibrate the initial structures of the adsorbates' layer and not to thoroughly explore the configurational space. Furthermore, we are only interested in the rearrangements at the surface, which can explain the observed loss of the ordered

superstructure of bidentate formate upon co-adsorption of small amount of water. These rearrangements involve a limited number of degrees of freedom in our models. The large size of the system, i.e., the multi-layered oxide slab, is necessary to correctly reproduce the electronic properties and the interaction field.
The Metadynamics approach has been applied to reveal the mechanism that leads to the exchange of positions among adsorbed formate and water. Hence, the initial state is quite obviously the superstructure of the bidentate formate layer, as revealed also by diffraction, to which a certain amount of water molecules has been added. To simulate the process by metadynamics we first focused on some specific steps, identifying collective variables involving only very few degrees of freedom. Based on the results of these exploratory runs, we could set metadynamics simulations with more general variables, thus reducing the bias on the simulated mechanisms. Focusing on one single formate is acceptable, since in the initial state all formate adsorbates are equivalent. The second collective variable, instead, involves all the present water molecules, hence allows any rearrangement among them.
Once the metadynamics space has been defined by the selection of the collective variable, the metadynamics simulation is extended until convergence of the explored free energy surface in this space. We used quite conservative metadynamics settings, to guarantee a slow and thorough sampling of the CV-space. Our simulations have been repeatedly restarted until convergence of the free energy profile has been reached. The additional evidence of convergence is that, after the transition, the trajectory comes back towards the initial state. We are confident that our choice of CVs is meaningful, since we were able to capture the process back and forth (formate leaving the surface as formic acid, and later approaching it again) and the results are consistent with the experimental findings.
Regarding the C1s peak centered around 285 eV in Fig. 1 – we recently published a detailed study of the role of carbon contamination when exposing the TiO_2(110) system to liquid water and water vapor, including a detailed quantitative analysis. Therefore, we added a sentence: "A detailed analysis of the C 1s region under similar experimental conditions can be found in Ref. 15." [15. Comini, N.; Huthwelker, T.; Diulus, J. T.; Osterwalder, J.; Novotny, Z. Factors influencing surface carbon contamination in ambient-pressure x-ray photoelectron spectroscopy experiments. *J. Vac. Sci. Technol. A* **2021**, *39* (4), 043203.]
We concede that the term formate radical is inappropriate modified the main text and the SI, replacing it with the term 'formate' everywhere.

Reviewer: 2

Recommendation: This paper may be publishable, but major revision is needed; I would like to be invited to review any future revision.

Comments:
The authors present a new study on the dynamic equilibrium between water vapor and a rutile TiO2(110) surface saturated with a (2x1) overlayer of HCOOH. The authors present compelling evidence for the restructuring of a formate HCOO- monolayer on TiO2 by exposing a saturated surface with sequential dosing of water vapor.

I recommend publishing in J. Phys. Chem. Lett. with the following mandatory revisions listed below. I am also requesting to see the revised draft.

1. The dissociative chemisorption of formic acid on rutile $TiO_2(110)$ has been well-documented over the past 25 years. Throughout the manuscript, the authors refer to the chemisorbed formate anion ($HCOO^-$) as a “formate radical”. This is incorrect. Formic acid dissociatively chemisorbs on the $TiO_2(110)$ as a formate anion to undercoordinated Ti^{4+} and the proton on the bridging oxygen. The undercoordinated Ti^{4+} sites act as a Lewis Acid, and the bridging oxygen as a Lewis Base.
The article, as written, must be changed to reflect the correct anionic character of the formate species. Dissociative chemisorption of carboxylic acids on TiO_2 is not a radical-based mechanism.

Related issues: In the abstract, the authors state that the bidentate binds “bidentate way”. This sounds rather strange; please reformulate and remove the word “way”. Also, on page 12, lines 21-23, the authors state that “The hydronium ion then interacts with the formate radical, where a hydrogen atom hops over to form a formic acid molecule.” Formally, it is a proton that is transferred, not hydrogen atoms. Please correct.

2. In the introduction, the authors write, “While in principle, different adsorption geometries are in principle possible…” Previous researchers have observed monodentate species.[1]

3. The adventitious carbon XPS peak is much larger than that of HCOO carbon. The relative coverages of both species should be quantified. The authors state that coverage of HCOO carbon is calculated to be ~0.5 ML. This is inconsistent with the large adventitious C seen in Figure 1. It is also surprising that such a large amount of the adventitious C would allow for the formation of the (2×1) phase in LEED. Please explain how such a large amount of adventitious C can coexist with the ordered (2×1) phase.

4. Figure 2 shows processed LEED images of saturated HCOOH-terminated surfaces after sequential exposure to H_2O vapor. Please provide corresponding XPS C1s data from water exposures. Very little is discussed with respect to the relationship between the LEED changes and the chemistry that occurs on the surface. High-resolution scans of the C1s region would provide this information and increase the impact of this manuscript. The levels of adventitious C in such data should also be provided. Additionally, the time-dependent stability of the (2x1) overlayer under the x-ray and e- beams should be discussed.

5. Several published manuscripts show that water rinses self-assembled monolayers off.[2] Please discuss the prior studies and the factors (kinetics, solvation) that are known to lead to washing off the self-assembled monolayers.

6. When describing Figure S1, the authors state on pg. 8 that panel (a) shows a rutile surface fully covered with formic acid. The description should state that the surface is covered by bidentate hydroxyl pairs. Additionally, please add another panel showing molecularly-bound

formic acid and its energetics. This will complement the discussion and illustrate the relative binding energy of molecularly-bound water and formic acid species. Further, please include the per-molecule binding energies in the figure or the caption.

7. Figures S2 and S3 – include the per-molecule binding energies in the figure or the caption.

8. Page 9, paragraph 2 – list the temperature and water pressure used in the BOMD simulations.

9. In the discussion of Figure 4, it is not clear whether the listed values of the free energies refer to the exergonicity of the steps or to their barriers. Both values are important and should be listed for each elementary step. Further, free energies are usually indicated using the symbol G, not E, and relative values are indicated by using the symbol delta. Please list the values of free energies in panels A-F. The last panel in Figure 4 presents the 2-dimensional free energy diagram. For clarity and the benefit of the readers, it would be very useful to graphically present the lowest free energy path along the reaction coordinate with the set of elementary steps.

10. Having the free energy dependencies should provide a straightforward path to construct the pressure and temperature-dependent kinetics of formate removal—something the authors could consider.

11. List the experimental temperature on page 4, line 2. On line 3, page 4, list the colors of the spectra that are being referred to in the text.

References

1. A. Mattsson, S. Hu, K. Hermansson, L Osterlund, "Adsorption of formic acid on rutile TiO2(110) revisited: An infrared reflection-adsorption spectroscopy and density functional theory study" J. Chem. Phys. 140, 034705 (2014).
2. E. Skibinski, W. J. I. DeBenedetti, and M. A. Hines, "Solution Deposition of Phenylphosphinic Acid Leads to Highly Ordered, Covalently Bound Monolayers on TiO2(110) Without Annealing" J. Phys. Chem. C 121 14213-14221 (2017).

Reply:

1. We recognize that the description of the adsorbed species was not appropriate. In the revised version of the manuscript the term 'formate' is used to indicate the dissociative chemisorption of formic acid. Following the Reviewer's suggestion, we removed the word 'way' from the abstract, and also corrected the sentence on page 12 (lines 21-23). It now reads: "The hydronium ion then interacts with the desorbing formate, where a proton hops over to form a formic acid molecule".

2. The sentence has been improved. It now reads: "While different adsorption geometries are possible,[12] this is the most favorable one, [13] as was previously verified by x-ray photoelectron diffraction (XPD), [14] STM[15-16] and infrared reflection-absorption spectroscopy.[12]"

3. A quantification of the 285 eV C 1s peak area with respect to the formate peak is now included. Also, we added a half sentence discussing the form of adventitious carbon (being in a form of large clusters), which is also compatible with the sharp substrate spots we observe in LEED after water exposure. The modified paragraph now reads as:

The spectra are dominated by an "adventitious" carbon peak at 285.0 eV, related to C-C bonds, which is unavoidable in this pressure range. A detailed analysis of the C 1s region under similar experimental conditions can be found in Ref. 17. Before water exposure a distinctive peak is observed at 289.3 eV, commonly associated with surface formate.[10] Calculating the formate coverage with a thin film model[18] yields an approximate 0.5 ML coverage, consistent with a fully saturated TiO_2(110) surface, while the peak area of the "adventitious" carbon peak is 6.1±0.7 times higher than the formate peak. The saturation coverage of formate indicates that the "adventitious" carbon does not uniformly cover the surface, but is rather present in the form of large clusters. After water exposure this peak is replaced by a broader and weaker shoulder that is commonly present as the "adventitious" carbon contamination increases (having a 17±2 times larger area compared to the initial formate peak).[17] This suggests that adsorbed formate molecules are displaced under exposure to water vapor.

4. We added a sentence on page four, where we provide a reference to a paper where we studied carbon contamination on TiO_2(110) when exposed to water (liquid water, water vapor). The sentence reads as:

A detailed analysis of the C 1s region under similar experimental conditions can be found in Ref. 16.

In addition, we addressed the stability of the formate layer under electron irradiation by extending the last paragraph on page six:

"After a chosen time, the gate valve was re-opened to quickly return to HV where LEED patterns were measured in the preparation chamber using commercial low-current LEED optics to avoid electron-induced degradation of the formate overlayer caused by the measurement.[20] To reduce the effect of carbon contamination (as shown in Fig. 1), the analysis chamber was exposed to repeated cycles of water exposure (mbar range) followed by pumping to HV, which progressively reduced the amount of "adventitious" carbon.[17]"

To address the time-dependent stability of the (2x1) overlayer during LEED measurements, we added the following sentence to the Experimental details (SI):

"The sample was irradiated with electrons only during LEED acquisition (4 seconds) when a mechanical shutter was opened. Test measurements were performed, rendering the influence of electron-stimulated desorption negligible due to the electron beam flux being ~50× lower than conventional LEED instruments."

To address the time-dependent stability of the (2x1) overlayer when measured with XPS, we added the following sentences to the end of the last paragraph on page 4:

"While the feasibility of rinsing the ordered monolayers due to their high solubility in water was reported previously,[10, 19] the XPS also highlighted the use of high-intensity synchrotron x-ray beam inadequate. Much of the "adventitious" carbon buildup is a result of the exposure to the x-ray beam.[17] In addition, the secondary electrons produced by the XPS technique are known to induce a rapid degradation of the ordered formate overlayer.[20]"

Finally, we fully agree with Reviewer that the complementary C 1s spectra would complete the story. Unfortunately, such measurements are not suitable for synchrotron-based measurements due to the focused, high-intensity x-ray beam, which is notoriously known to build up carbon on the sample. Our setup currently does not have a laboratory X-ray anode source to irradiate a large area on the sample that would allow such measurements. This is why we have used low-current MCP LEED. While we could use the LEED optics as an AES spectrometer, such measurements use a rather high electron beam current (typically above 30 uA), which will distort the formate overlayer within seconds, as reported in the literature.
We currently have no access to a laboratory-based XPS setup equipped with a high-pressure cell for gas dosing since the Osterwalder group (responsible for the experimental part) was dissolved six months ago. We will have the possibility to acquire the additional C1s spectra only in 3.5 months when the last author of this manuscript (ZN) starts his work at PNNL where he will have such instruments available, but this would mean we will need around 5 months from now to provide the corresponding C 1s dataset.

5. We added a half-sentence on page 4 highlighting that the rinsing of ordered, self-assembled overlayers was reported previously:

While the feasibility of rinsing the ordered monolayers due to their high solubility in water was reported previously, [9, 18] the XPS also highlighted the use of high-intensity synchrotron x-ray beam inadequate.

In addition, we added the following sentence to the introduction on page 2: "The presence of an ultrathin water layer can change the reaction energetics significantly, as was previously demonstrated for a spontaneous reaction of atmospheric CO_2 with the nanoscale film of H_2O adsorbed to the rutile (110) surface.[8]"

6. We thank the Reviewer for the remark. We have changed the description of Figure S1 in the main text, and we now state that the surface is covered by 'bidentate formate-hydroxyl pairs'. We appreciate the Reviewer's suggestion of showing the molecularly-bound formic acid and its energetics in Figure S1. We have also added a brief discussion including these values to the main text.
The per molecule binding energies for the cases shown in panels B and C of Figure S1 are already presented in the main text (page 8), but we also added them to the caption of Figure S1.

7. For Figure S2, we have already provided the water adsorption energies in the main text. In the revised version of the manuscript, they have also been added to the caption of the figure. We have also added the average per molecule bidentate formate-hydroxyl pair adsorption energy

for the structure shown in panel A of Figure S2. In the case of Figure S3, the instantaneous configurations extracted from BOMD trajectories are illustrated. Then obviously it makes no sense to compare the instantaneous potential energies.

8. The water pressure in our model is not well defined, because the simulation cell includes the~10Å vacuum layer in the z-direction. Water molecules have been added to provide the optimal coverage and to saturate the hydrogen bonds within the first layer by a second layer. The resulting model was equilibrated for 10 ps so, where the equipartition of the average kinetic energy per degree of freedom corresponding to 300 K is over the entire system.

9. We apologize for the misunderstanding and thank Reviewer 2 for the comment. The values we are reporting are the free energy barriers, and we have already corrected that in the main text. Moreover, we have also modified both the main text and the SI, and we now refer to the relative free energy values as ΔG. We decided for not adding the free energy values to panels A-F of Figure 4, since with this extra information the figure becomes too cramped. Finally, we have also added the lowest free energy path to the 2D free energy surface.
10. This study was meant to disclose the mechanism of the structural rearrangement occurring upon co-adsorption of formic acid and water at the oxide surface. We can conclude that a small amount of water is sufficient to induce the re-structuring. However, the pressure-temperature phase diagram is out of the scope of the submitted manuscript.

11. We added the experimental temperature (room temperature (297 ± 2 K)) on page 3, line 2 of the original manuscript. The colors of the spectra in Fig. 1 are now referenced in the text on page 4.

Reviewer: 3

Recommendation: This paper is publishable subject to minor revisions noted. Further review is not needed.

Comments:
1. What is the major advance reported in the paper?

The study uses a combination of experimental and theoretical methods to examine the interaction of water with an adsorbed formate layer on rutile TiO_2 (110). Photoemission measurements provide convincing evidence that dipping in water causes formate to be desorbed, with LEED results being consistent with this finding. The theoretical section details a reaction mechanism in which the water aids the desorption of the bound formate species.

2. What is the immediate significance of this advance?

This paper presents a challenge to a previous study (Balajka et al, Science 2018--ref 9) that concluded that formate binds preferentially over water (and other adsorbates) on rutile, creating

a hydrophobic surface. The key difference highlighted is the effect of chemical equilibria on formate adsorption, such that the partial pressure of formic acid in air and/or solution concentration will play a key role in determining the nature of the contact layer. This is critical for an understanding in a number of applications, such as self-cleaning glass.

Given the major finding of the work, it important to correct an inaccuracy in the manuscript introduction regarding refs 8 and 9. Ref 8 concluded that OH was formed at the contact layer after dipping and immersing the (110) substrate in water. This is not accurately described as 'restructuring' as stated in the manuscript. Ref 9 re-interpreted these earlier results as formate in the contact layer based on the supposed displacement of water by formate, and the assumption that there was a small concentration of the acid in the ultra-pure water employed in the earlier work. The results of the current work rule out the latter interpretation, which is a significant finding that should be brought out more in the discussion section.

Reply: We thank the Reviewer for a positive reception of our manuscript. We agree that the term "restructuring" when relating to Ref. 8 is inappropriate. We added that the study performed by the Diebold group (Ref. 9 in the original manuscript, Ref. 10 in the revised one) was performed with oxygen-free water since, indeed, the results of Ref. 9 could not be explained by the presence of a small amount of formic acid that would be not compatible with the quantitative analysis of the C coverage being only 0.1 ML. We modified the respective section in the introduction, which now reads as:
"Early studies on a clean TiO_2(110) surface suggested that liquid water could form a (2×1) periodicity due to the ordered array of hydroxyl molecules with a liquid water in the second layer.[9] Later scanning tunneling microscopy (STM) and x-ray photoelectron spectroscopy (XPS) experiments demonstrated that ultrapure, oxygen-free water itself is not responsible for the observed surface (2×1) periodicity.[10]"

We still keep the term restructuring in place on page 10 where we discuss the MTD simulations and restructuring relates to the formate adsorbates, not the TiO_2 surface.

3. Technical suggestions

The dipping procedure should be described in the current manuscript (SI).

Reply: The following sentence was added to the Experimental details section in the SI:
"Sample was dipped into liquid water using the dip-and-pull technique (for more details, see Refs. 1. 2)."

A low current LEED apparatus was employed, but was a test carried out to ensure the absence of beam damage?

Reply: We are fully aware of the electron-stimulated desorption (ESD) reported previously in the literature. Tests were performed to assess the electron beam effect on our measurements. We could see a similar spot intensity ratio when increasing the water exposure step-wise, or when

the same total water exposure was performed in a single step. The following sentences were added to the Experimental details section (SI):
"The sample was irradiated with electrons only during LEED acquisition (4 seconds) when a mechanical shutter was opened. Test measurements were performed, rendering the influence of electron-stimulated desorption negligible due to the electron beam being ~50× lower than conventional LEED instruments."

It is not clear how many water molecules were included in the MD simulations. Is it the number shown in the movie?

Reply: We apologize for the fact that the information about the number of water molecules added was not easily found by the Referee. In the SI (page 3), we state that we added 11 and 9 extra water molecules to structures 1 and 2, respectively. This information is also mentioned in the caption of Figure S3, where structures 1 and 2 are shown.

jz-2022-03788e.R2

Name: Peer Review Information for "Dynamic equilibrium at the HCOOH-saturated TiO_2(110)-water interface"

Second Round of Reviewer Comments

Reviewer: 2

Comments to the Author

While the authors have made progress in addressing my concerns, I still find the issue of adventitious carbon lacking clarity. It seems that when three-dimensional carbon clusters are present, most X-ray photons (due to the glancing angle) must penetrate through these clusters. As such, they disproportionally contribute to the carbon 1s signal. However, the paper does not provide a sufficient explanation of this phenomenon. This issue needs to be clearly explained in the paper. After the authors expand on this issue in the manuscript, the article can be accepted for publication in the Journal of Physical Chemistry Letters.

Author's Response to Peer Review Comments:

All responses are included in the attached DOCX file

Reply to the editor: All non-scientific changes have been performed.

Reviewer(s)' Comments to Author:

Reviewer: 2

Recommendation: This paper is publishable subject to minor revisions noted. Further review is not needed.

Comments:
While the authors have made progress in addressing my concerns, I still find the issue of adventitious carbon lacking clarity. It seems that when three-dimensional carbon clusters are present, most X-ray photons (due to the glancing angle) must penetrate through these clusters. As such, they disproportionally contribute to the carbon 1s signal. However, the paper does not provide a sufficient explanation of this phenomenon. This issue needs to be clearly explained in the paper. After the authors expand on this issue in the manuscript, the article can be accepted for publication in the Journal of Physical Chemistry Letters.

Reply: We thank Reviewer for a positive reception of our manuscript. Following the suggestion of the Reviewer, we have added an extra sentence on page 4, where we further discuss the origin of a large C 1s signal in the spectra shown in Fig. 1. The added sentence reads as follows:

"Since most x-ray photons penetrate through these clusters due to the glancing incidence angle, they disproportionally contribute the C 1s signal located at 285 eV in Fig. 1."

Due to the added sentence, we also modified the following sentence, where the energy of the formate peak was indicated. The modified sentence now reads as:

"After water exposure, the 289.3 eV formate peak is replaced by a broader and weaker shoulder that is commonly present as the “adventitious” carbon contamination increases (having a 17±2 times larger area compared to the initial formate peak).[17]"

Additional Questions:
Urgency: Moderate

Significance: High

Novelty: High

Scholarly Presentation: Moderate

Is the paper likely to interest a substantial number of physical chemists, not just specialists working in the authors' area of research?: Yes

Additional changes not indicated by the Reviewers or Editors:

- We realized that reference 9 and reference 10 were assigned incorrectly in the SI document on page S3 of the Supplementary Information document. We have swapped references 9 and 10.

- One of the co-authors (J.T.D.) realized that one of his founding sources is not acknowledged. We deeply apologize for this. As a consequence, the following sentence was added in the Acknowledgment section in the main manuscript:

  "JTD acknowledges funding from European Union’s Horizon 2020 under MCSA Grant No 801459, FP-RESOMUS."

- In the SI document, on page S5, we found that we still used the term "bidentate way" and "monodentate way" when referring to the adsorption of formic acid molecules. This was pointed out in the first review by both Reviewer 1 and Reviewer 2. The term "way" has been removed and formulated in a proper manner.
